# Supplementary material for: Urbanization and cardiovascular health among Indigenous groups in Brazil
Source: Commun Med (Lond). 2023 Feb 2;3:17. doi: 10.1038/s43856-023-00239-3 (PMC9895427; doi:10.1038/s43856-023-00239-3)
Supplement: Supplementary file 3 — Description of Additional Supplementary Files [file 43856_2023_239_MOESM3_ESM.pdf]

Description of Additional Supplementary Files

Supplementary Data 1 - Clinical parameters for Fulni-ô Indigenous people, Truká Indigenous people and urban non-Indigenous population. Brazil.

Legend: BMI – body mass index; NC- neck circumference; WC - waist circumference; HC – hip circumference; WHR - waist-to-hip ratio; SBD – systolic blood pressure; DBP – diastolic blood pressure; MAP – mean arterial pressure; ABI - Ankle-brachial index· [(1)] chi-squared continuity correction; [(1a)] chi-squared continuity correction (sex comparasion); [(2)] Kuskal-Wallis test.

Supplementary Data 2 - Source data for Figure 2.

Legend: Total population (n = 999): Fulni-ô People (n = 303; 30.3%); the intermediate Truká Indigenous group (n = 336; 33.7%); highly urbanized cohort living in the city of Juazeiro (n = 360; 36.0%). *Control group refers to the urban non-Indigenous population
