# Supplementary material for: Urbanization and cardiovascular health among Indigenous groups in Brazil
Source: Commun Med (Lond). 2023 Feb 2;3:17. doi: 10.1038/s43856-023-00239-3 (PMC9895427; doi:10.1038/s43856-023-00239-3)
Supplement: Supplementary file 5 — Reporting Summary [file 43856_2023_239_MOESM5_ESM.pdf]

nature research

Corresponding author(s): DBPR COMMSMED-22-0640A

Last updated by author(s): Jan 3, 2023

Reporting Summary

Nature Research wishes to improve the reproducibility of the work that we publish. This form provides structure for consistency and transparency in reporting. For further information on Nature Research policies, see our Editorial Policies and the Editorial Policy Checklist.

Statistics

For all statistical analyses, confirm that the following items are present in the figure legend, table legend, main text, or Methods section.

n/a	Confirmed	
☐	☒	The exact sample size (n) for each experimental group/condition, given as a discrete number and unit of measurement
☐	☒	A statement on whether measurements were taken from distinct samples or whether the same sample was measured repeatedly
☐	☒	The statistical test(s) used AND whether they are one- or two-sided<br>*Only common tests should be described solely by name; describe more complex techniques in the Methods section.*
☐	☒	A description of all covariates tested
☐	☒	A description of any assumptions or corrections, such as tests of normality and adjustment for multiple comparisons
☐	☒	A full description of the statistical parameters including central tendency (e.g. means) or other basic estimates (e.g. regression coefficient) AND variation (e.g. standard deviation) or associated estimates of uncertainty (e.g. confidence intervals)
☐	☒	For null hypothesis testing, the test statistic (e.g. F, t, r) with confidence intervals, effect sizes, degrees of freedom and P value noted<br>*Give P values as exact values whenever suitable.*
☒	☐	For Bayesian analysis, information on the choice of priors and Markov chain Monte Carlo settings
☒	☐	For hierarchical and complex designs, identification of the appropriate level for tests and full reporting of outcomes
☐	☒	Estimates of effect sizes (e.g. Cohen's d, Pearson's r), indicating how they were calculated

Our web collection on statistics for biologists contains articles on many of the points above.

Software and code

Policy information about availability of computer code

Data collection	Open source software from Rega Institute for Medical Research, customized for this study: REGA-PAI.
Data analysis	JASP (Jeffreys's Amazing Statistics Program) version 0.16.1 (Department of Psychological Methods, University of Amsterdam, Amsterdam, The Netherlands); QGis veriosn 2.14.11 (Open Source Geospatial Foundation, Beaverton, OR, USA).

For manuscripts utilizing custom algorithms or software that are central to the research but not yet described in published literature, software must be made available to editors and reviewers. We strongly encourage code deposition in a community repository (e.g. GitHub). See the Nature Research guidelines for submitting code & software for further information.

Data

Policy information about availability of data

All manuscripts must include a data availability statement. This statement should provide the following information, where applicable:

- Accession codes, unique identifiers, or web links for publicly available datasets
- A list of figures that have associated raw data
- A description of any restrictions on data availability

Data on Brazilian indigenous peoples are restricted by several regulations. Therefore, we are not allowed to freely distribute our dataset. A researcher interested in accessing our dataset must seek authorization from the official regulatory agency: Fundação Nacional do Índio – Funai (https://www.gov.br/funai/pt-br).

Field-specific reporting

Please select the one below that is the best fit for your research. If you are not sure, read the appropriate sections before making your selection.

☒ Life sciences ☐ Behavioural & social sciences ☐ Ecological, evolutionary & environmental sciences

For a reference copy of the document with all sections, see nature.com/documents/nr-reporting-summary-flat.pdf

Life sciences study design

All studies must disclose on these points even when the disclosure is negative.

Sample size	All indigenous people living in the Fulni-ô an Truká communities were invited to participate in the PAI study in advance, using community meetings, indigenous leadership communications, and the available media. The urban non-Indigenous population was recruited in the city of Juazeiro, Bahia. Neighborhoods in Juazeiro with a low migration profile were visited by the PAI staff and the habitants were invited to participate by community meetings and local political leadership communications. All invited individuals that met inclusion criteria and were willing to participate were included, after providing a written consent.
Data exclusions	No data excluded from the analysis.
Replication	No experimental findings in this manuscript.
Randomization	No experimental groups in this study.
Blinding	Not applicable

Reporting for specific materials, systems and methods

We require information from authors about some types of materials, experimental systems and methods used in many studies. Here, indicate whether each material, system or method listed is relevant to your study. If you are not sure if a list item applies to your research, read the appropriate section before selecting a response.

Materials & experimental systems

n/a	Involved in the study	
☒	☐	Antibodies
☒	☐	Eukaryotic cell lines
☒	☐	Palaeontology and archaeology
☒	☐	Animals and other organisms
☐	☐	Human research participants
☒	☐	Clinical data
☒	☐	Dual use research of concern

Methods

n/a	Involved in the study	
☒	☐	ChIP-seq
☒	☐	Flow cytometry
☒	☐	MRI-based neuroimaging

Human research participants

Policy information about studies involving human research participants

Population characteristics	we included women and men aged between 30 and 70 years old, residents of the Indigenous Fulni-ô and Truká communities or in the city of Juazeiro. We excluded those with clinically manifested heart failure, history of coronary or cerebrovascular vascular diseases requiring hospitalization, renal failure on dialysis, history of surgery for peripheral arterial disease or heart disease, and participants who expressed reluctancy to undergo diagnostic tests including blood draw.
Recruitment	All indigenous people living in the Fulni-ô an Truká communities were invited to participate in the PAI study in advance, using community meetings, indigenous leadership communications, and the available media. The urban non-Indigenous population was recruited in the city of Juazeiro, Bahia. Neighborhoods in Juazeiro with a low migration profile were visited by the PAI staff and the habitants were invited to participate by community meetings and local political leadership communications. All invited individuals that met inclusion criteria and were willing to participate were included, after providing a written consent.
Ethics oversight	The PAI study was approved by the National Research Ethics Council (CONEP number 1.488.268), the National Indigenous Foundation (Fundação Nacional do Índio [FUNAI]; process number 08620.028965/2015-66), and the Indigenous leaders of both participating groups. All participants provided written informed consent before enrollment in the study.

Note that full information on the approval of the study protocol must also be provided in the manuscript.
